# The impact of IGRA positivity in untreated inactive pulmonary tuberculosis on IVF-ET outcomes in infertile women: an ambispective cohort study

**DOI:** 10.3389/fmed.2025.1626519

**Published:** 2025-07-24

**Authors:** Xiaoyan Gai, Hongbin Chi, Lin Zeng, Lixue Chen, Zikang Sheng, Wenli Cao, Chen Zhang, Jingge Qu, Yue Zhang, Weixia Zhang, Qingtao Zhou, Ping Liu, Yongchang Sun, Rong Li, Jie Qiao

**Affiliations:** ^1^Department of Respiratory and Critical Care Medicine, Peking University Third Hospital, Beijing, China; ^2^Center for Reproductive Medicine, Department of Obstetrics and Gynecology, Peking University Third Hospital, National Clinical Research Center for Obstetrics and Gynecology, Beijing, China; ^3^Clinical Epidemiology Research Center, Peking University Third Hospital, Beijing, China; ^4^Tuberculosis Department, Beijing Geriatric Hospital, Beijing, China; ^5^Information Center, Peking University Third Hospital, Beijing, China

**Keywords:** interferon-gamma release assay, tuberculosis, *in vitro* fertilization and embryo transfer, pregnancy outcome, infertility

## Abstract

**Background:**

Tuberculosis can negatively impact both overall health and female reproductive function. This study investigated the relationship between the interferon-gamma release assay (IGRA) status and pregnancy outcomes in infertile women, with untreated “inactive” tuberculosis lesions observed on chest radiography, who are undergoing *in vitro* fertilization and embryo transfer (IVF-ET).

**Methods:**

This ambispective cohort study, which includes retrospective (2012–2019) and prospective (2020–2024) cohorts, enrolled infertile women with untreated inactive tuberculosis lesions visible on chest imaging who are planning to undergo IVF-ET. All patients underwent IGRA testing. Baseline characteristics, such as age, body mass index (BMI), infertility factors, ultrasound follicle count, and hormone levels, were collected. Pregnancy outcomes, including live birth rates, oocyte retrieval numbers, embryo quality, clinical pregnancy, miscarriage, and preterm birth rates, were followed and compared between the IGRA-positive and IGRA-negative groups.

**Results:**

Among 836 patients, the IGRA positivity rate was 42.5%. The cumulative miscarriage rate was higher in the IGRA-positive group than in the IGRA-negative group (21.5% vs. 15.0%, *p* = 0.047). No significant differences were found in clinical pregnancy or live birth rates. Age, BMI, and endometrial thickness were independent risk factors influencing clinical pregnancy and live birth rates, while the IGRA status was not.

**Conclusion:**

In infertile women with untreated inactive tuberculosis lesions on chest radiography, IGRA positivity is associated with higher cumulative miscarriage rates following IVF-ET. Early IGRA screening and intervention may help improve pregnancy outcomes.

## Graphical abstract

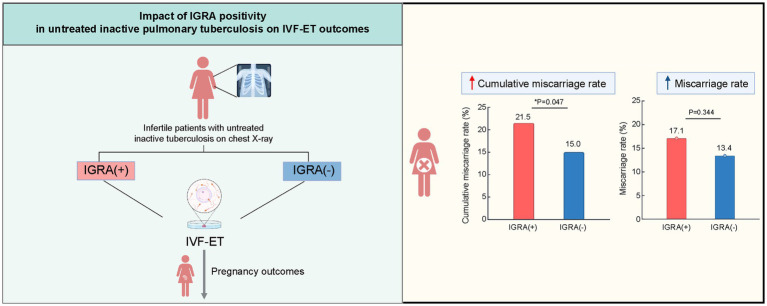

## 1 Introduction

Tuberculosis (TB) remains a significant global public health issue (1). Among infertile patients, TB is a common cause of infertility (2, 3), particularly in high-burden countries, accounting for 20–30% of infertility cases. TB can lead to infertility through tubal obstruction, endometrial involvement, and decreased ovarian function (4).

*In vitro* fertilization and embryo transfer (IVF-ET) are effective for managing infertility, and routine chest radiography is recommended before undergoing IVF-ET (3). “Inactive” pulmonary TB lesions, like calcified nodules, observed on chest imaging, are often overlooked. In our previous retrospective cohort study, patients with untreated inactive pulmonary TB lesions experienced significantly lower clinical pregnancy and live birth rates following IVF-ET compared to those without such lesions (5). Additionally, our research team reported seven cases of miliary TB during pregnancy following IVF-ET, identifying primary infertility, inactive TB lesions visible on chest radiographs, and tubal obstruction as common risk factors (6). Therefore, patients experiencing infertility and showing inactive TB lesions on chest radiographs should receive comprehensive TB screening prior to IVF-ET, coupled with careful management during pregnancy (7).

Latent tuberculosis infection (LTBI) is characterized by a sustained immune response to *Mycobacterium tuberculosis* antigens without progression to active TB disease (8–10). Treating LTBI is crucial for the “End TB Strategy” (10). LTBI is usually diagnosed using a positive tuberculin skin test (TST) or interferon-gamma release assay (IGRA). The TST may yield false positives due to prior Bacillus Calmette–Guérin (BCG) vaccination, while IGRA demonstrates higher specificity (9). Guidelines recommend preventive anti-TB treatment for individuals at high risk of TB, including those with acquired immunodeficiency syndrome, those on corticosteroids or immunosuppressive therapy, and organ transplant recipients (11). However, the impact of LTBI on pregnancy outcomes following IVF-ET remains unclear (12, 13), and there are no established guidelines or expert consensus on whether patients with infertility and LTBI should receive preventive anti-TB treatment.

Therefore, this study aimed to address two key scientific questions: (1) What proportion of infertile women with untreated inactive TB lesions on chest radiographs are IGRA-positive? (2) Does IGRA positivity affect pregnancy outcomes following IVF-ET? The results of this study could provide valuable evidence to guide clinical practice (Clinical Trial: NCT 04443283). This study was registered as a clinical trial (NCT 04443283) in 2019, and the protocol has been published elsewhere (12).

## 2 Methods

### 2.1 Study design and population

This study was conducted at Peking University Third Hospital, a tertiary care hospital located in Beijing, China. An ambispective cohort study design was employed, enrolling patients with infertility undergoing IVF-ET at our reproductive center. The study was conducted in two phases: a prospective phase from January 2020 to September 2023 and a retrospective phase from January 2012 to December 2019. Patients were screened according to Chinese guidelines for infertility diagnosis and treatment, which recommend chest radiography screening before IVF-ET (14).

Patients whose chest radiographs showed “inactive” TB lesions (e.g., fibrotic nodules and calcifications detailed in Supplementary material S1) (15) and had no history of anti-TB treatment were included, as described in our published protocol (7). Those with active TB or who met other exclusion criteria, such as central nervous system disease, cancer, or acquired immunodeficiency syndrome, were excluded.

The study was approved by the Ethics Committee of Peking University Third Hospital (No. IRB00006761-M2020244). Written informed consent was obtained from all participants in the prospective cohort. For the retrospective cohort, informed consent was waived by the Ethics Committee as the data were anonymized and de-identified.

### 2.2 Grouping of latent tuberculosis infection

Here, IGRA positivity was used as the standard criterion for defining LTBI (7). Participants were assigned to the IGRA-positive and IGRA-negative groups. Two common methods for IGRA testing include T-SPOT. TB (Oxford Immunotec Ltd., Abingdon, UK) and QuantiFERON®-TB Gold (QFT; Qiagen, Hilden, Germany). Between January 2012 and December 2019, our center used the T-SPOT. TB method, in which Panel A or B spot counts >6 were considered positive. The QFT method was used from January 2020 onward, where QFT > 0.35 was considered positive. Our laboratory confirmed the consistency between the QFT and T-SPOT. TB results from a previous study (16) (Supplementary material S2).

### 2.3 *In vitro* fertilization and embryo transfer treatment procedure

The IVF-ET procedure was performed in adherence to strict standard operating procedures for ovarian stimulation, oocyte retrieval, *in vitro* fertilization, and embryo transfer, as detailed in previous reports from our center (17). Eligible participants underwent one of four IVF-ET protocols: long, short, ultralong, or antagonist. After oocyte retrieval, IVF was performed, and 1–2 high-quality embryos were selected for transfer on the third- or fifth-day post-retrieval, following the regulations of the Chinese Ministry of Health. Following ET, progesterone vaginal gel (90 mg) was administered daily until 10 weeks of gestation. Serum human chorionic gonadotropin (HCG) levels were measured 14 days and 21 days post-transfer. Ultrasonography was conducted at 30- and 37-days post-transfer to assess the presence of a gestational sac and fetal heartbeat.

### 2.4 Primary and secondary outcome measures

The primary outcome was the live birth rate following IVF-ET in the IGRA-positive and IGRA-negative groups. Secondary outcomes included the clinical pregnancy and miscarriage rates. IVF-ET treatment characteristics, such as number of oocytes retrieved, endometrial thickness, and the rate of high-quality embryos, were also assessed. Additionally, the cumulative pregnancy, live birth, and miscarriage rates were tracked for each participant until July 30, 2024.

The primary outcome measures and various rates were calculated as follows: clinical pregnancy rate = (number of clinical pregnancy cycles/number of transfer cycles) × 100%; miscarriage rate = (number of early miscarriages + number of late miscarriages)/number of clinical pregnancies × 100%; and live birth rate = (number of live birth cycles/number of transfer cycles) × 100%. The cumulative pregnancy, live birth, and miscarriage rates during the study period were calculated for each participant based on the proportion of cumulative clinical pregnancies, live births, or miscarriages over ≥1 transfer cycles. The denominator was the total number of cases for each participant. Further details on outcome definitions, calculation methods, and embryo grading can be found in Supplementary material S3.

### 2.5 Data collection

Baseline data, including age, body mass index (BMI), duration of infertility, and ovarian stimulation protocols, were collected from all patients. The levels of follicle-stimulating hormone, luteinizing hormone, estradiol, progesterone, prolactin, testosterone, androgen, and anti-Müllerian hormone were recorded. Additionally, ultrasound findings of the endometrium, the number of follicles, HCG levels, and ultrasound results, such as the presence of a gestational sac and endometrial thickness, were documented after ovarian stimulation.

For the retrospective cohort, relevant data were retrieved from hospital medical records. The specialized follow-up team of our reproductive center monitored pregnancy outcomes for up to 1 year for each patient who underwent IVF-ET. Additionally, information on the TB status, anti-TB treatment, and TB-related symptoms during pregnancy was collected through medical record review and follow-up telephone calls by the research team from our respiratory department.

### 2.6 Sample size calculations

To assess the effect of IGRA positivity on pregnancy outcomes, we considered several variables, including age, BMI, causes of infertility, controlled ovarian hyperstimulation (COH) protocol, endometrial thickness, and number of high-quality embryos. Following the general guideline for multivariate logistic regression, which suggests a minimum of 10 events per variable, we estimated that 190 live birth events would be needed. Assuming a live birth rate of 30%, a total sample of 600 participants who underwent ET was required to achieve adequate statistical power.

### 2.7 Statistical analysis

All statistical analyses were performed using SPSS for Windows, version 25 (IBM Corporation, Armonk, NY, USA). Continuous data are expressed as mean±standard deviation (x ± s) and were compared between groups using the independent samples *t*-test or the Mann–Whitney U test. Categorical data are expressed as frequencies and percentages and were compared using the chi-square test. Pregnancy outcomes, including live birth, clinical pregnancy, and miscarriage rates, were compared between the IGRA-positive and IGRA-negative groups using the chi-squared test. Multivariate analysis of pregnancy outcomes was performed using logistic regression, with the results reported as odds ratios (ORs), 95% confidence intervals (CIs), and *p*-values. Statistical significance was set at *p* < 0.05.

## 3 Results

### 3.1 Baseline characteristics

**Figure 1:**
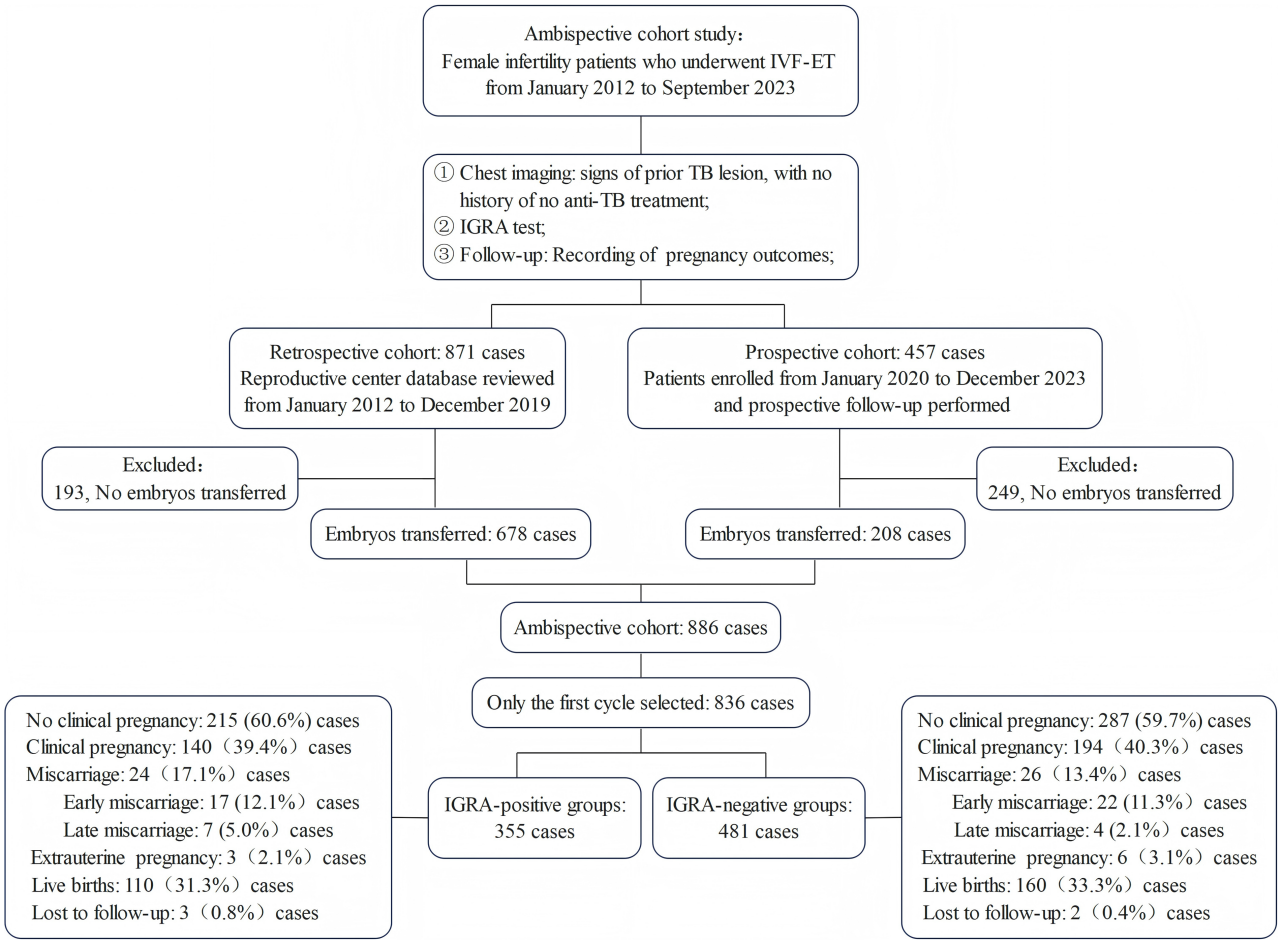
Flowchart of the ambispective cohort study. This diagram outlines the selection of female infertility patients who underwent IVF-ET from 2012 to 2023, divided into IGRA-positive and IGRA-negative groups. The prospective cohort includes patients from 2020 to 2023, while the retrospective cohort covers those from 2012 to 2019. The flowchart summarizes the primary pregnancy outcomes, including clinical pregnancy, miscarriage, and live birth. TB, tuberculosis; IVF-ET, *in vitro* fertilization and embryo transfer; IGRA, interferon-gamma release assay.

Overall, 886 eligible participants who underwent IVF-ET were enrolled, including 678 from the retrospective cohort and 208 from the prospective cohort. The outcomes of 836 first fresh cycles were analyzed. Among patients with untreated inactive TB lesions detected on chest radiography, the LTBI positivity rate was 42.5% (355/836). When comparing baseline characteristics, IGRA-positive patients were slightly younger (mean age, 33.79 ± 4.93 vs. 34.01 ± 4.44 years, *p* = 0.091), had a higher BMI (mean, 22.28 ± 3.24 vs. 22.29 ± 4.00 kg/m^2^, *p* = 0.008), and had a longer infertility duration (mean, 4.84 ± 4.00 vs. 4.13 ± 3.18 years, *p* < 0.001) than IGRA-negative patients. Significant differences in the causes of infertility were found between the groups (*p* = 0.001), with a higher proportion of tubal factor infertility and ovulatory disorders in the IGRA-positive group than in the IGRA-negative group (29.9% vs. 24.1 and 9.3% vs. 4.6%, respectively) (Figure 1; Table 1).

**Table 1:** Characteristics of patients with infertility and untreated pulmonary tuberculosis.

Characteristic	IGRA-positive group (*n* = 355)	IGRA-negative group (*n* = 481)	*p*
Age (years)	33.79 ± 4.93	34.01 ± 4.44	0.091
Body mass index (kg/m^2^)	22.28 ± 3.24	22.29 ± 4.00	0.008*
Duration of infertility (years)	4.84 ± 4.00	4.13 ± 3.18	<0.001*
Causes of infertility, number (%)			0.001*
Fallopian tube factor	106 (29.9)	116 (24.1)	
Ovulation disorders	33 (9.3)	22 (4.6)	
Endometriosis	5 (1.4)	11 (2.3)	
Polycystic ovary syndrome	11 (3.1)	16 (3.3)	
Oligospermia/asthenospermia	35 (9.9)	80 (16.6)	
Unexplained infertility	32 (9.0)	34 (7.1)	
Mixed female factors	24 (6.8)	23 (4.8)	
Mixed both sexes	91 (25.6)	133 (27.7)	
Other female factors	3 (0.8)	20 (4.2)	
Other male factors	15 (4.2)	26 (5.4)	
Basic hormone levels			
FSH, mIU/mL	6.63 (5.33–8.41)	6.54 (4.80–8.35)	0.271
LH, mIU/mL	3.46 (2.16–4.60)	3.18 (2.16–4.84)	0.651
E2, pg./mL	156.00 (116.00–217.00)	152.00 (115.00–202.25)	0.389
P, nmol/L	1.14 (0.81–1.55)	1.16 (0.88–1.49)	0.727
AMH, ng/mL	1.98 (0.82–3.34)	1.91 (1.08–3.09)	0.552

FSH, follicle-stimulating hormone; LH, luteinizing hormone; E2, estradiol; P, progesterone; PRL, prolactin; T, testosterone; AMH, anti-Müllerian hormone; IGRA, interferon-gamma release assay; IVF-ET, in vitro fertilization and embryo transfer; Data on FSH, LH, E2, P, and AMH were missing for 22 cases, 39 cases, 47 cases, 43 cases, and 354 cases respectively, in the general research population (AMH levels test has been implemented since 2017). *p < 0.05.

### 3.2 *In vitro* fertilization and embryo transfer treatment characteristics

No significant differences were found between the IGRA-positive and IGRA-negative groups in IVF-ET treatment characteristics, including COH protocols, endometrial thickness, number of oocytes retrieved, high-quality embryo rate, and number of embryos transferred (Table 2).

**Table 2:** Protocols of COH and data of IVF-ET.

Characteristic	IGRA-positive group (*n* = 355)	IGRA-negative group (*n* = 481)	*p*
Protocol of COH, number (%)			0.499
Ultralong GnRH agonist	39 (10.9)	50 (10.4)	
Short GnRH agonist	12 (3.3)	20 (4.2)	
GnRH antagonist	200 (56.3)	253 (52.6)	
Microsimulation cycle	8 (2.2)	20 (4.2)	
Long GnRH agonist	96 (27.0)	137 (28.5)	
Natural or other	0 (0)	1 (0.2)	
Endometrial thickness (mm)	10.59 ± 1.78	10.65 ± 1.70	0.750
Number of retrieved oocytes per cycle, median (IQR)	9 (6–13)	9 (6–13)	0.916
Number of good-quality embryos per cycle, median (IQR)	4 (2–6)	3 (2–6)	0.742
Number of embryos transferred			0.632
1	45 (12.6)	73 (15.2)	
2	193 (54.3)	283 (58.8)	
Number of embryo transfer cycles	2.0 (1.0–3.0)	2.0 (1.0–3.0)	0.773

COH, controlled ovarian hyperstimulation; IQR, interquartile range; GnRH, gonadotropin-releasing hormone; IGRA, interferon-gamma release assay; IVF-ET, in vitro fertilization and embryo transfer.

### 3.3 Interferon-gamma release assay and single-cycle pregnancy outcomes

The IGRA-positive group showed a trend toward a higher miscarriage rate than the IGRA-negative group (17.1% vs. 13.4%), although the difference was not significant (*p* = 0.344). No difference was found in preterm birth rate between the IGRA-positive group and the IGRA-negative group (10.7% vs. 11.9%, *p* = 0.746). Among the 24 miscarriage cases in the IGRA-positive group, two women experienced spontaneous miscarriages caused by active miliary pulmonary TB during pregnancy, and they recovered after anti-TB therapy. Additionally, miscarriage occurred in 26 cases in the IGRA-negative group, with no TB cases during pregnancy. The clinical pregnancy rates in the IGRA-positive and IGRA-negative groups were 39.4 and 40.3%, respectively, and the live birth rates were 31.0 and 33.3%, respectively; no significant differences were observed (Table 3).

**Table 3:** IVF-ET pregnancy outcomes in a single fresh cycle of IVF-ET in patients who were IGRA positive and IGRA negative.

Outcome	Group (*n*)	Rate (%)	Univariate analysis		Multivariate analysis	
			OR (95% CI)	*p*	aOR (95% CI)	*p*
Clinical pregnancy rate	IGRA-positive group (*n* = 355)	39.4% (140/355)	0.963 (0.728–1.275)	0.794	1.026 (0.672–1.564)	0.907
	IGRA-negative group (*n* = 481)	40.3% (194/481)				
Live birth rate	IGRA-positive group (*n* = 355)	31.0% (110/355)	0.901 (0.671–1.209)	0.486	0.951 (0.609–1.484)	0.824
	IGRA-negative group (*n* = 481)	33.3% (160/481)				
Miscarriage rate	IGRA-positive group (*n* = 140)	17.1% (24/140)	1.337 (0.731–2.444)	0.344	1.066 (0.425–2.674)	0.891
	IGRA-negative group (*n* = 194)	13.4% (26/194)				

aOR, adjusted odds ratio; CI, confidence interval; IGRA, interferon-gamma release assay; IVF-ET, in vitro fertilization and embryo transfer.

Multivariate regression analysis, which included IGRA positivity, age, BMI, infertility factors, endometrial thickness, COH protocols, and the number of good-quality embryos as independent variables and pregnancy outcomes of the first fresh cycle post-enrollment as dependent variables, revealed that age was a significant factor influencing the clinical pregnancy rates (41–45 years vs. ≤30 years, OR = 0.331, 95% CI: 0.121–0.909, *p* = 0.032). BMI and endometrial thickness were also identified as significant factors influencing the clinical pregnancy and live birth rates. BMI was negatively associated with the clinical pregnancy (OR = 0.912, 95% CI: 0.852–0.975, *p* = 0.007) and live birth (OR = 0.907, 95% CI: 0.844–0.974, *p* = 0.008) rates, while endometrial thickness was positively associated with both outcomes (clinical pregnancy: OR = 1.307, 95% CI: 1.147–1.489, *p* < 0.001; live birth: OR = 1.332, 95% CI: 1.161–1.529, *p* < 0.001). IGRA positivity was not an independent risk factor for the clinical pregnancy, live birth, or miscarriage rates (Table 3; Supplementary Table S1).

### 3.4 Interferon-gamma release assay and cumulative pregnancy, cumulative miscarriage, and cumulative live birth rates

The cumulative miscarriage rate was significantly higher in the IGRA-positive group than in the IGRA-negative group (21.5% vs. 15.0%, *p* = 0.047). However, no significant differences in the cumulative clinical pregnancy [66.8% vs. 67.8% (*p* = 0.757)] and cumulative live birth [56.3% vs. 58.4% (*p* = 0.564)] rates were found between the IGRA-positive and IGRA-negative groups.

Multivariate regression analysis, which included IGRA positivity, age, BMI, infertility factors, endometrial thickness, COH protocols, the number of good-quality embryos, and the number of embryo transfer cycles as independent variables and cumulative pregnancy outcomes as dependent variables, revealed that IGRA positivity was not significantly associated with the cumulative clinical pregnancy, cumulative live birth, or cumulative miscarriage rates (Table 4; Supplementary Table S2).

**Table 4:** Cumulative pregnancy outcomes of patients who were IGRA positive and IGRA negative.

Outcome	Group (*n*)	Rate (%)	Univariate analysis		Multivariate analysis	
			OR (95% CI)	*p*	aOR (95% CI)	*p*
Cumulative pregnancy rate	IGRA-positive group (*n* = 355)	66.8%	0.955 (0.713–1.279)	0.757	0.942 (0.609–1.456)	0.788
	IGRA-negative group (*n* = 481)	67.8%				
Cumulative live birth rate	IGRA-positive group (*n* = 355)	56.3%	0.918 (0.696–1.212)	0.547	0.923 (0.615–1.386)	0.699
	IGRA-negative group (*n* = 481)	58.4%				
Cumulative miscarriage rate	IGRA-positive group (*n* = 237)	21.5%	1.550 (1.004–2.392)	**0.047**^**a**^	1.247 (0.675–2.307)	0.481
	IGRA-negative group (*n* = 326)	15.0%				

aOR, adjusted odds ratio; IGRA, interferon-gamma release assay; ^a^*p* < 0.05. Bold values indicate statistical significance (*p* < 0.05).

## 4 Discussion

This study investigated whether IGRA positivity affects pregnancy outcomes in women with infertility and untreated “inactive” TB lesions seen on chest radiographs, a population that has been mostly overlooked. Our cohort study showed that patients who were IGRA positive had a significantly higher cumulative miscarriage rate. Additionally, a trend toward a higher miscarriage rate in the first fresh cycle was observed in the IGRA-positive group, although this difference was not statistically significant. These findings suggest that LTBI may increase the risk of miscarriage in women undergoing IVF-ET.

Previous studies have indicated that LTBI may impact pregnancy outcomes through mechanisms such as impaired ovarian function, difficulties in embryo implantation, or altered immune responses (3, 18). LTBI has also been associated with chronic low-grade systemic inflammation and dysregulation of cytokine networks, including alterations in the balance of immune cell subsets, such as Th1, Th2, and Th17 cells (19). Disruptions in the endometrial immune microenvironment may further contribute to adverse pregnancy outcomes (20). Further research is needed to elucidate these underlying mechanisms in this population.

Currently, no guidelines recommend routine LTBI screening for patients with infertility and untreated “inactive” TB lesions seen on chest radiographs. In our previous study, 10.4% of patients with infertility seeking assisted reproduction had untreated “inactive” TB lesions on chest radiographs (5). In the present study, the IGRA positivity rate was 42.5% in this population, significantly higher than the LTBI prevalence of 18.1% in the general rural Chinese population (9) and higher than the 28.6% rate in individuals with radiographically inactive TB who lacked a history of TB treatment in rural China (21). Previous studies have reported a latent risk of reactivation associated with untreated inactive TB in the general population (22–24). Patients with infertility, in particular, should be closely monitored for potential TB reactivation during pregnancy (5, 25).

Previous studies on LTBI and pregnancy outcomes following IVF-ET have shown mixed results. One study reported no significant differences in pregnancy or neonatal outcomes between the TST-positive and TST-negative groups (26). Another retrospective study found that the IGRA-positive group had lower biochemical pregnancy rates, lower clinical pregnancy rates, and lower live birth rates than the IGRA-negative group, indicating a trend toward reduced outcomes, although no statistically significant differences (13). Further, a small cohort study in the United States involving 323 women with infertility found that 7.7% (25/323) of them were diagnosed with LTBI via blood IGRA and that the rates of recurrent spontaneous abortion (28% vs. 7%) and Asherman syndrome (8% vs. 0.3%) were significantly higher in women with LTBI than in those without (27). Inconsistent results across studies may stem from variations in LTBI diagnostic methods and the small sample sizes of ET studies. Our research uniquely focused on patients with untreated, “inactive” TB lesions seen on chest radiographs. We found an increased cumulative miscarriage rate as well as a trend toward higher single-cycle miscarriage rates in the IGRA-positive group than in the IGRA-negative group. However, IGRA was not identified as an independent factor in multivariate analysis,; this may be due to the small number of miscarriage events. Given the limited total miscarriage cases and analysis of multiple factors (including age, BMI, infertility factors, ovarian stimulation protocol, IGRA status, endometrial thickness, and high-quality embryo rate), false-negative results may have been produced. Therefore, further studies with larger sample sizes are needed to confirm these findings.

Furthermore, in the present study, two patients in the IGRA-positive group experienced miliary TB during pregnancy following IVF-ET, underscoring the potential risk of TB reactivation and its profound implications for maternal and fetal health, including miscarriage, stillbirth, or low birth weight. Women undergoing IVF-ET experience immune modulation due to hormonal stimulation and the immune-suppressive environment required for embryo implantation. These alterations in immune function may compromise the body’s ability to contain latent TB infections, thereby increasing the risk of reactivation during pregnancy (10). Preventive anti-TB treatment for such patients with infertility may therefore offer benefits by reducing the risk of TB reactivation.

Some experts in the United States and Canada have recommended that members of the general population with nodular or fibrotic lesions consistent with old TB are high-priority candidates for preventive anti-TB treatment of LTBI after active TB disease is excluded (22–24). However, a recent randomized controlled trial in the general population in rural China found that a 6-week preventive treatment regimen for 677 individuals with IGRA positivity and radiographically inactive TB lesions did not significantly reduce the incidence of active TB (21). Studies specifically focused on populations with infertility remain limited.

The global incidence of infertility among women is as high as 10–15%, and an increasing number of reports of TB during pregnancy have recently emerged in high TB burden in countries, such as China (5, 25), and also in developed countries, primarily among immigrants from countries with high TB prevalence (28). A study in India on latent female genital TB found that after anti-TB treatment, the implantation and clinical pregnancy rates were higher in the latent TB group than in the non-TB group. In contrast, the miscarriage rate was lower (29). However, preventive anti-TB treatment may delay assisted reproduction, which is particularly problematic for older women. Freezing embryos and preserving fertility before initiating preventive anti-TB treatment, followed by thawed ET, could offer a solution. In clinical practice, the side effects of anti-TB medications, including liver toxicity and potential adverse effects on the fetus, also need to be considered. Therefore, neither the international guidelines on LTBI nor the guidelines on infertility diagnosis and treatment currently recommend preventive anti-TB treatment before IVF-ET (30). However, a recent expert consensus in China suggested that LTBI screening and preventive anti-TB treatment for patients with infertility undergoing assisted reproduction may be helpful to improve pregnancy outcomes (31). Future randomized controlled trials are needed to assess the risks and benefits of preventive anti-TB treatment, thereby providing evidence-based guidance for the development of guidelines.

This study has several limitations. First, this was a single-center study; however, as the National Clinical Research Center for Obstetrics and Gynecology and one of the largest reproductive centers in China, our center serves a diverse patient population nationwide, enhancing the representativeness of the sample. Additionally, the IGRA testing and IVF-ET treatment protocols at our center are homogeneous and subject to strict quality control. Second, we only analyzed the impact of IGRA results on pregnancy outcomes, not of the TST results, although the TST is more commonly used in primary care. However, because TST results can be affected by the Bacillus Calmette–Guérin vaccination, IGRA is regarded as a more accurate measure of LTBI. Finally, while we excluded patients with active TB, routine laparoscopy was not performed for all participants, leaving the possibility of latent genital TB undetermined.

## 5 Conclusion

This study demonstrated that, among Chinese women with infertility and untreated “inactive” TB lesions seen on chest radiographs undergoing IVF-ET, the IGRA positivity rate reached 42.5%, with a higher cumulative miscarriage rate following IVF-ET observed among patients who were IGRA positive. These results underscore the importance of considering LTBI screening and management in clinical practice, particularly in regions with high TB prevalence. Future research should prioritize larger, multicenter studies to investigate further the effectiveness of preventive anti-TB treatment in improving fertility treatment success rates.

## Data Availability

The original contributions presented in the study are included in the article/Supplementary material, further inquiries can be directed to the corresponding authors.
