# Supplementary material for: The impact of IGRA positivity in untreated inactive pulmonary tuberculosis on IVF-ET outcomes in infertile women: an ambispective cohort study
Source: Front Med (Lausanne). 2025 Jul 24;12:1626519. doi: 10.3389/fmed.2025.1626519 (PMC12328282; doi:10.3389/fmed.2025.1626519)
Supplement: Supplementary file 1 [file Supplementary_file_1.docx]

**Supplementary Material**

**Table of Contents**

| Supplementary Data 1. Chest Imaging Analysis | 2 |
| --- | --- |
| Supplementary Data 2. Validation of QuantiFERON (QFT) Method Consistency with T-SPOT.TB | 2 |
| Supplementary Data 3. Primary Outcome Measures | 4 |
| Supplementary Table 1. Results of multivariate regression analysis of pregnancy outcomes in a single fresh cycle of IVF-ET | 6 |
| Supplementary Table 2. Results of multivariate regression analysis of cumulative pregnancy outcomes in IVF-ET | 10 |

**Supplementary Data 1. Chest Imaging Analysis**

Inactive TB lesions on chest X-rays are characterized as clear-bordered linear opacities; irregularly shaped fibrotic nodules, particularly those with calcification; secondary bronchiectasis; and pleural thickening, all of which were suggestive of inactive TB in this study^1,2^ Other pulmonary diseases, including silicosis and sarcoidosis, were excluded.

Active pulmonary TB on chest X-rays is characterized by patchy or lobar consolidation, nodular lesions, cavities, tuberculomas, irregular mass-like shadows, bronchogenic dissemination, hilar lymphadenopathy, and pleural effusion. These findings were suggestive of active TB, which was excluded from the study.

Chest X-ray findings were confirmed by a respiratory physician and a radiologist.

**Supplementary Data 2. Validation of QuantiFERON (QFT) Method Consistency with T-SPOT.TB**

To ensure consistency between the QFT and T-SPOT.TB methods, a validation study was conducted by the Department of Laboratory Medicine at Peking University Third Hospital during the transition to the QFT method in January 2020. Fifty samples were tested using both methods in a double-blind manner. The results showed a 92% consistency rate, exceeding the required threshold of 85%. This indicates that the QFT method provides reliable results comparable with those provided by the T-SPOT.TB method. This validation was personally supervised and reviewed by Dr. Cui Liyan, Director of the Department of Laboratory Medicine, ensuring the accuracy and reliability of the results.

**Evaluation Report on the Mycobacterium Tuberculosis-Specific Cellular Immune Response Test Kit (QFT)**

**Objective**

To conduct a technical evaluation of the ongoing *Mycobacterium tuberculosis*-specific cellular immune response test to assess whether the test kit meets the laboratory’s analytical performance requirements.

**Methods**

1. Instruments:

 37°C ± 1°C dry incubator (no CO₂ required)

 Dual-wavelength microplate reader: 450-nm filter, with auxiliary filter between 620 and 650 nm

 Centrifuge (2000–3000 RCF(g))

1. **Reagents**:

 *Mycobacterium tuberculosis*-specific cellular immune response test kit (ELISA), Product Registration Number: GuoXieZhuJin 20173406702

1. **Sample type:**

 Whole blood

1. **Experimental method**

 Comparison with T-SPOT:

Fifty samples were collected, and both T-SPOT and QFT tests were performed in a double-blind manner. The results of the two methods were compared to calculate the consistency rate between the two tests. A consistency rate of ≥85% was required.

**Results**

The comparison between T-SPOT and QFT demonstrated a 92% consistency rate, surpassing the required threshold of 85%. Thus, this validation confirmed that the *Mycobacterium tuberculosis*-specific cellular immune response test meets laboratory performance standards. The results were consistent with other testing methods and deemed reliable for clinical diagnostics, meeting the demands of routine clinical practice.

We also conducted a comprehensive review of existing literature and meta-analyses, as summarized below. The findings demonstrate comparable sensitivity for active TB detection across diverse populations, and high concordance for LTBI detection.

**Supplementary Literature: Performance of T-SPOT.TB vs. QFT-GIT in Active TB and LTBI**

| **Population** | **Metric** | **T-SPOT** | **QFT-GIT** | **Ref.** |
| --- | --- | --- | --- | --- |
| ***Active TB*** |  |  |  |  |
| Unspecified | Sensitivity | 84-96.9% | 84.2-97.9% | [1-3] |
|  | Specificity | 65.8-100% | 74.5-98.1% |  |
| HIV+ patients | Sensitivity | 60-89% | 66-69% | [4-5] |
|  | Specificity | 86-87% | 76-87% |  |
| Elderly | Sensitivity | 68.1% | 91.4% | [6] |
|  | Specificity | 100% | 100% |  |
| Children | Sensitivity | 87-88.5% | 83-89.6% | [7-8] |
|  | Specificity | 96.8-99% | 93-95.4% |  |
| ***LTBI*** |  |  |  |  |
| High-risk | Concordance | κ = 0.71 | | [9] |
|  | Positive agreement | 91% | |  |
| Immunosuppressed | Concordance | κ =0.53 | | [10] |
|  | Positive agreement | 83.2% | |  |

Reference

1. Zhang, Yu et al. “Comparing the diagnostic performance of QuantiFERON-TB Gold Plus with QFT-GIT, T-SPOT.TB and TST: a systematic review and meta-analysis.” *BMC infectious diseases* vol. 23,1 40. 20 Jan. 2023, doi:10.1186/s12879-023-08008-2
2. Takasaki, Jin et al. “Sensitivity and specificity of QuantiFERON-TB Gold Plus compared with QuantiFERON-TB Gold In-Tube and T-SPOT.TB on active tuberculosis in Japan.” *Journal of infection and chemotherapy : official journal of the Japan Society of Chemotherapy* vol. 24,3 (2018): 188-192. doi:10.1016/j.jiac.2017.10.009
3. Lu, Peng et al. “Interferon-Gamma Release Assays for the Diagnosis of Tuberculosis: A Systematic Review and Meta-analysis.” *Lung* vol. 194,3 (2016): 447-58. doi:10.1007/s00408-016-9872-5
4. Chen H, Nakagawa A, Takamori M, et al. Diagnostic accuracy of the interferon-gamma release assay in acquired immunodeficiency syndrome patients with suspected tuberculosis infection: a meta-analysis. *Infection*. 2022;50(3):597-606. doi:10.1007/s15010-022-01789-9
5. Huo, Zhen-Yu, and Li Peng. “Accuracy of the interferon-γ release assay for the diagnosis of active tuberculosis among HIV-seropositive individuals: a systematic review and meta-analysis.” *BMC infectious diseases* vol. 16 350. 22 Jul. 2016, doi:10.1186/s12879-016-1687-8
6. Fukushima, Kiyoyasu et al. “Clinical evaluation of QuantiFERON®-TB Gold Plus directly compared with QuantiFERON®-TB Gold In-Tube and T-Spot®.TB for active pulmonary tuberculosis in the elderly.” *Journal of infection and chemotherapy : official journal of the Japan Society of Chemotherapy* vol. 27,12 (2021): 1716-1722. doi:10.1016/j.jiac.2021.08.016
7. Laurenti, Patrizia et al. “Performance of interferon-γ release assays in the diagnosis of confirmed active tuberculosis in immunocompetent children: a new systematic review and meta-analysis.” *BMC infectious diseases* vol. 16 131. 18 Mar. 2016, doi:10.1186/s12879-016-1461-y
8. Hirabayashi, Ryosuke et al. “Utility of interferon-gamma releasing assay for the diagnosis of active tuberculosis in children: A systematic review and meta-analysis.” *Journal of infection and chemotherapy : official journal of the Japan Society of Chemotherapy* vol. 30,6 (2024): 516-525. doi:10.1016/j.jiac.2023.12.007
9. Venkatappa, Thara K et al. “Comparing QuantiFERON-TB Gold Plus with Other Tests To Diagnose Mycobacterium tuberculosis Infection.” *Journal of clinical microbiology* vol. 57,11 e00985-19. 23 Oct. 2019, doi:10.1128/JCM.00985-19
10. Fernández-Blázquez, Ana et al. “Diagnosis of Tuberculous Infection in Immunosuppressed Patients and/or Candidates for Biologics Using a Combination of 2 IGRA Tests: T-SPOT.TB/QuantiFERON TB Gold In-Tube vs. T-SPOT.TB/QuantiFERON TB Gold Plus.” “Diagnóstico de la infección tuberculosa en pacientes inmunodeprimidos y/o candidatos a terapias biológicas mediante el uso combinado de dos pruebas IGRA: T-SPOT.TB/QuantiFERON TB Gold In-Tube vs. T-SPOT.TB/QuantiFERON TB Gold Plus.” *Archivos de bronconeumologia* vol. 58,4 (2022): 305-310. doi:10.1016/j.arbres.2020.04.011

**Supplementary Data 3. Primary Outcome Measures**

The pregnancy outcomes of patients in the IGRA-positive and IGRA-negative groups were monitored following *in vitro* fertilization and embryo transfer (IVF-ET). The primary outcome was the live birth rate following IVF-ET.

The secondary outcomes included clinical pregnancy and miscarriage following IVF-ET. Clinical pregnancy, miscarriage, and live birth rates were analyzed based on the outcomes of the first fresh cycle for each participant. We also observed the IVF-ET treatment characteristics, including ovarian stimulation protocols, the number of oocytes retrieved, endometrial thickness, and the rate of high-quality embryos. Moreover, the cumulative pregnancy, live birth, and miscarriage rates were analyzed for each participant from the time of enrollment until 30 July 2024.

The primary outcome measures and various rates were calculated as follows: clinical pregnancy rate = (number of clinical pregnancy cycles / number of transfer cycles) × 100%; miscarriage rate = (number of early miscarriages + number of late miscarriages) / number of clinical pregnancies × 100%; and live birth rate = (number of live birth cycles / number of transfer cycles) × 100%. According to the PETER cleavage stage embryo grading system, embryos with 6–10 blastomeres on day 3 graded as I or II were defined as high-quality embryos, whereas the remainder were classified as non-high-quality embryos. High-quality embryo rate = (number of high-quality embryos / number of fertilized eggs) × 100%.

The cumulative pregnancy, live birth, and miscarriage rates during the study period were calculated for each participant based on the proportion of cumulative clinical pregnancies, live births, or miscarriages over ≥1 transfer cycle. The denominator was the total number of cases for each participant. For example, if a participant had no live births by the final cycle, their outcome was considered as no live birth.

**References**

1 Gai XY, Chi HB, Zeng L, et al. Impact of positive interferon-gamma release assay on IVF-ET pregnancy outcomes in infertile patients with untreated prior tuberculosis: a prospective cohort study. Front Med (Lausanne) 2021; **8**:749410.

2 Hicks A, Muthukumarasamy S, Maxwell D, Howlett D. Chronic inactive pulmonary tuberculosis and treatment sequelae: chest radiographic features. Int J Tuberc Lung Dis 2014; **18**:128–33.

3 陈立雪,李蓉,叶荣伟.中国妇女体外受精-胚胎移植累计活产率分析[J].中国生育健康杂志, 2017, 28(2):5.DOI:10.3969/j.issn.1671-878X.2017.02.001.

Chen Lixue, Li rong, Ye rongwei. Cummulative live birth rate in vitro fertilization in China: A population-based study. Chinese Reproductive Health, 2017, 28(2):5.DOI:10.3969/j.issn.1671-878X.2017.02.001.（Chinese）

**Supplementary Table 1. Results of multivariate regression analysis of pregnancy outcomes in a single fresh cycle of IVF-ET**

| **Pregnancy outcomes** | **OR** | **95% CI** | **P value** |
| --- | --- | --- | --- |
| **Clinical pregnancy rate (n=836)** |  |  |  |
| LTBI (+) vs. LTBI (-) | 1.026 | 0.672–1.564 | 0.907 |
| Age (years) |  |  | 0.312 |
| 31–35 vs. £30 | 0.791 | 0.468–1.337 | 0.382 |
| 36–40 vs. £30 | 0.711 | 0.380–1.331 | 0.286 |
| 41–45 vs. £30 | 0.331 | 0.121–0.909 | 0.032^*^ |
| ≥46 vs. £30 | —— | —— | 0.999 |
| BMI (kg/m^2^) | 0.912 | 0.852–0.975 | 0.007^*^ |
| Endometrial thickness | 1.307 | 1.147–1.489 | <0.001^*^ |
| COH treatment protocol |  |  | 0.696 |
| Short protocol vs. ultra-long protocol | 0.537 | 0.143–2.015 | 0.356 |
| Antagonist protocol vs. ultra-long protocol | 0.956 | 0.480–1.901 | 0.897 |
| Minimal stimulation cycle vs. ultra-long protocol | 2.157 | 0.450–10.352 | 0.337 |
| Long protocol vs. ultra-long protocol | 0.968 | 0.469–2.000 | 0.931 |
| Natural or other vs. ultra-long protocol | —— | —— | —— |
| Cause of infertility |  |  | 0.256 |
| Endometriosis vs. tubal factor | 0.352 | 0.064–1.923 | 0.228 |
| PCOS vs. tubal factor | 0.558 | 0.137–2.262 | 0.414 |
| Ovulation disorder vs. tubal factor | 0.879 | 0.344–2.245 | 0.787 |
| Mixed female factors vs. tubal factor | 0.658 | 0.283–1.531 | 0.331 |
| Other female factors vs. tubal factor | 0.196 | 0.023–1.687 | 0.138 |
| Male oligo/asthenospermia vs. tubal factor | 0.594 | 0.260–1.354 | 0.215 |
| Other male factors vs. tubal factor | 0.271 | 0.029–2.549 | 0.253 |
| Mixed male and female factors vs. tubal factor | 1.345 | 0.788–2.296 | 0.277 |
| Unexplained vs. tubal factor | 0.655 | 0.299–1.436 | 0.297 |
| Number of good-quality embryos | 1.044 | 0.979–1.113 | 0.194 |
| **Miscarriage rate (n=334)** |  |  |  |
| LTBI (+) vs. LTBI (-) | 1.066 | 0.425–2.674 | 0.891 |
| Age (years) |  |  | 0.696 |
| 31–35 vs. £30 | 0.596 | 0.200–1.775 | 0.353 |
| 36–40 vs. £30 | 0.723 | 0.201–2.594 | 0.619 |
| 41–45 vs. £30 | 1.805 | 0.172–18.890 | 0.622 |
| ≥46 vs. £30 | —— | —— | —— |
| BMI (kg/m^2^) | 1.094 | 0.928–1.291 | 0.284 |
| Endometrial thickness | 0.909 | 0.665–1.243 | 0.549 |
| COH treatment protocol |  |  | 0.518 |
| Short protocol vs. ultra-long protocol | 1.128 | 0.021–60.723 | 0.953 |
| Antagonist protocol vs. ultra-long protocol | 6.148 | 0.619–61.048 | 0.121 |
| Minimal stimulation cycle vs. ultra-long protocol | —— | —— | 0.999 |
| Long protocol vs. ultra-long protocol | 4.358 | 0.403–47.166 | 0.226 |
| Natural or other vs. ultra-long protocol | —— | —— | —— |
| Cause of infertility |  |  | 0.340 |
| Endometriosis vs. tubal factor | —— | —— | 0.999 |
| PCOS vs. tubal factor | —— | —— | 0.999 |
| Ovulation disorder vs. tubal factor | 7.991 | 1.274–50.117 | 0.027^*^ |
| Mixed female factors vs. tubal factor | 0.449 | 0.048–4.172 | 0.481 |
| Other female factors vs. tubal factor | —— | —— | 1.000 |
| Male oligo/asthenospermia vs. tubal factor | 0.533 | 0.054–5.243 | 0.590 |
| Other male factors vs. tubal factor | —— | —— | 1.000 |
| Mixed male and female factors vs. tubal factor | 0.898 | 0.280–2.880 | 0.857 |
| Unexplained vs. tubal factor | 3.947 | 0.989–15.750 | 0.052 |
| Number of good-quality embryos | 1.021 | 0.874–1.192 | 0.793 |
| **Live birth rate (n=831)** |  |  |  |
| LTBI (+) vs. LTBI (-) | 0.951 | 0.609–1.484 | 0.824 |
| Age (years) |  |  | 0.292 |
| 31–35 vs. £30 | 0.900 | 0.522–1.550 | 0.704 |
| 36–40 vs. £30 | 0.696 | 0.357–1.357 | 0.287 |
| 41–45 vs. £30 | 0.304 | 0.099–0.940 | 0.039^*^ |
| ≥46 vs. £30 | —— | —— | 0.999 |
| BMI (kg/m^2^) | 0.907 | 0.844–0.974 | 0.008^*^ |
| Endometrial thickness | 1.332 | 1.161–1.529 | <0.001^*^ |
| COH treatment protocol |  |  | 0.466 |
| Short protocol vs. ultra-long protocol | 0.457 | 0.107–1.944 | 0.289 |
| Antagonist protocol vs. ultra-long protocol | 0.745 | 0.365–1.519 | 0.418 |
| Minimal stimulation cycle vs. ultra-long protocol | 2.366 | 0.480–11.652 | 0.290 |
| Long protocol vs. ultra-long protocol | 0.788 | 0.372–1.668 | 0.533 |
| Natural or other vs. ultra-long protocol | 1.332 | 1.161–1.529 | <0.001^*^ |
| Cause of infertility |  |  | 0.191 |
| Endometriosis vs. tubal factor | 0.173 | 0.019–1.569 | 0.119 |
| PCOS vs. tubal factor | 0.803 | 0.194–3.317 | 0.762 |
| Ovulation disorder vs. tubal factor | 0.565 | 0.188–1.696 | 0.309 |
| Mixed female factors vs. tubal factor | 0.867 | 0.363–2.071 | 0.749 |
| Other female factors vs. tubal factor | 0.267 | 0.030–2.344 | 0.233 |
| Male oligo/asthenospermia vs. tubal factor | 0.723 | 0.309–1.694 | 0.455 |
| Other male factors vs. tubal factor | 0.367 | 0.038–3.523 | 0.385 |
| Mixed male and female factors vs. tubal factor | 1.418 | 0.815–2.467 | 0.216 |
| Unexplained vs. tubal factor | 0.471 | 0.197–1.122 | 0.089 |
| Number of good-quality embryos | 1.023 | 0.957–1.094 | 0.506 |

Multivariate regression analysis was conducted using interferon-gamma release assay (IGRA) positivity/negativity, age, body mass index (BMI), infertility factors, endometrial thickness, controlled ovarian hyperstimulation (COH) protocols, and the number of good-quality embryos as independent variables, with the clinical pregnancy rate, live birth rate, and miscarriage rate as dependent variables. IVF-ET: *in vitro* fertilization and embryo transfer; CI: confidence interval; OR: odd ratio; IQR: interquartile range; LTBI: latent tuberculosis infection; PCOS: polycystic ovary syndrome.

**Supplementary Table 2. Results of multivariate regression analysis of cumulative pregnancy outcomes in IVF-ET**

| **Pregnancy outcomes** | **OR** | **95% CI** | **P value** |
| --- | --- | --- | --- |
| **Cumulative pregnancy rate (n=836)** |  |  |  |
| LTBI (+) vs. LTBI (-) | 0.942 | (0.609–1.456) | 0.788 |
| Age (years) |  |  | 0.421 |
| 31–35 vs. £30 | 1.138 | (0.654–1.981) | 0.648 |
| 36–40 vs. £30 | 1.099 | (0.574–2.106) | 0.775 |
| 41–45 vs. £30 | 0.835 | (0.347–2.011) | 0.688 |
| ≥46 vs. £30 | 0.100 | (0.008–1.277) | 0.076 |
| BMI (kg/m^2^) | 1.000 | (0.945–1.059) | 0.992 |
| Endometrial thickness | 1.051 | (0.929–1.188) | 0.433 |
| COH treatment protocol |  |  | 0.525 |
| Short protocol vs. ultra-long protocol | 0.464 | (0.148–1.453) | 0.187 |
| Antagonist protocol vs. ultra-long protocol | 1.152 | (0.577–2.298) | 0.688 |
| Minimal stimulation cycle vs. ultra-long protocol | 1.067 | (0.257–4.440) | 0.929 |
| Long protocol vs. ultra-long protocol | 1.147 | (0.550–2.393) | 0.715 |
| Natural or other vs. ultra-long protocol | —— | —— | —— |
| Cause of infertility |  |  | 0.340 |
| Endometriosis vs. tubal factor | 0.259 | (0.062–1.089) | 0.065 |
| PCOS vs. tubal factor | 0.535 | (0.167–1.722) | 0.295 |
| Ovulation disorder vs. tubal factor | 0.946 | (0.375–2.386) | 0.907 |
| Mixed female factors vs. tubal factor | 0.626 | (0.273–1.437) | 0.270 |
| Other female factors vs. tubal factor | 1.412 | (0.270–7.391) | 0.683 |
| Male oligo/asthenospermia vs. tubal factor | 0.361 | (0.163–0.799) | 0.012* |
| Other male factors vs. tubal factor | 0.611 | (0.101–3.710) | 0.592 |
| Mixed male and female factors vs. tubal factor | 0.727 | (0.409–1.292) | 0.277 |
| Unexplained vs. tubal factor | 0.691 | (0.297–1.612) | 0.393 |
| Number of good-quality embryos | 1.033 | (0.963–1.109) | 0.364 |
| Number of embryo transfer cycles | 1.037 | (0.858–1.253) | 0.710 |
| **Cumulative miscarriage rate (n=334)** |  |  |  |
| LTBI (+) vs. LTBI (-) | 1.247 | (0.675-2.307) | 0.481 |
| Age (years) |  |  | 0.483 |
| 31–35 vs. £30 | 0.533 | (0.247–1.150) | 0.109 |
| 36–40 vs. £30 | 0.634 | (0.254–1.579) | 0.327 |
| 41–45 vs. £30 | 1.194 | (0.321–4.447) | 0.791 |
| ≥46 vs. £30 | 0.464 | (0.930–1.075) | 1.000 |
| BMI (kg/m^2^) | 1.000 | —— | 1.000 |
| Endometrial thickness | 0.856 | (0.706–1.036) | 0.110 |
| COH treatment protocol |  |  | 0.638 |
| Short protocol vs. ultra-long protocol | 1.165 | (0.158–8.601) | 0.881 |
| Antagonist protocol vs. ultra-long protocol | 1.208 | (0.410–3.558) | 0.732 |
| Minimal stimulation cycle vs. ultra-long protocol | —— | —— | 0.999 |
| Long protocol vs. ultra-long protocol | 1.987 | (0.650–6.070) | 0.228 |
| Natural or other vs. ultra-long protocol | —— | —— | —— |
| Cause of infertility |  |  | 0.977 |
| Endometriosis vs. tubal factor | 2.203 | (0.165–29.444) | 0.551 |
| PCOS vs. tubal factor | 1.632 | (0.316–8.424) | 0.559 |
| Ovulation disorder vs. tubal factor | 1.354 | (0.413–4.443) | 0.617 |
| Mixed female factors vs. tubal factor | 0.527 | (0.128–2.163) | 0.374 |
| Other female factors vs. tubal factor | —— | —— | 0.999 |
| Male oligo/asthenospermia vs. tubal factor | 1.086 | —— | 0.900 |
| Other male factors vs. tubal factor | —— | —— | 0.999 |
| Mixed male and female factors vs. tubal factor | 0.993 | (0.436–2.261) | 0.987 |
| Unexplained vs. tubal factor | 1.552 | (0.531–4.534) | 0.422 |
| Number of good-quality embryos | 0.957 | (0.868–1.055) | 0.376 |
| Number of embryo transfer cycles | 1.485 | (1.149–1.920) | 0.003* |
| **Cumulative live birth rate (n=831)** |  |  |  |
| LTBI (+) vs. LTBI (-) | 0.923 | (0.615–1.386) | 0.699 |
| Age (years) |  |  | 0.890 |
| 31–35 vs. £30 | 1.284 | (0.768–2.147) | 0.341 |
| 36–40 vs. £30 | 1.140 | (0.623–2.086) | 0.670 |
| 41–45 vs. £30 | 0.988 | (0.424–2.306) | 0.979 |
| ≥46 vs. £30 | —— | —— | 0.999 |
| BMI (kg/m^2^) | 1.000 | (0.947–1.055) | 0.987 |
| Endometrial thickness | 1.118 | (0.994–1.257) | 0.063 |
| COH treatment protocol |  |  | 0.588 |
| Short protocol vs. ultra-long protocol | 0.430 | (0.136–1.359) | 0.151 |
| Antagonist protocol vs. ultra-long protocol | 0.971 | (0.502–1.878) | 0.929 |
| Minimal stimulation cycle vs. ultra-long protocol | 1.320 | (0.313–5.569) | 0.706 |
| Long protocol vs. ultra-long protocol | 0.898 | (0.446–1.806) | 0.762 |
| Natural or other vs. ultra-long protocol | —— | —— | —— |
| Cause of infertility |  |  | 0.369 |
| Endometriosis vs. tubal factor | 0.248 | (0.056–1.110) | 0.068 |
| PCOS vs. tubal factor | 0.556 | (0.179–1.726) | 0.310 |
| Ovulation disorder vs. tubal factor | 1.085 | (0.457–2.572) | 0.854 |
| Mixed female factors vs. tubal factor | 0.969 | (0.438–2.142) | 0.938 |
| Other female factors vs. tubal factor | 2.511 | (0.488–12.936) | 0.271 |
| Male oligo/asthenospermia vs. tubal factor | 0.456 | (0.210–0.988) | 0.047* |
| Other male factors vs. tubal factor | 1.239 | (0.204–7.512) | 0.816 |
| Mixed male and female factors vs. tubal factor | 0.786 | (0.464–1.333) | 0.372 |
| Unexplained vs. tubal factor | 0.785 | (0.361–1.710) | 0.543 |
| Number of good-quality embryos | 1.053 | (0.986–1.124) | 0.124 |
| Number of embryo transfer cycles | 0.933 | (0.784–1.110) | 0.431 |

Multivariate regression analysis was conducted using interferon-gamma release assay (IGRA) positivity/negativity, age, body mass index (BMI), infertility factors, endometrial thickness, controlled ovarian hyperstimulation (COH) protocols, the number of good-quality embryos and the number of embryo transfer cycles as independent variables, with the cumulative clinical pregnancy rate, cumulative live birth rate, and cumulative miscarriage rate as dependent variables. IVF-ET: *in vitro* fertilization and embryo transfer; CI: confidence interval; OR: odd ratio; IQR: interquartile range; LTBI: latent tuberculosis infection; PCOS: polycystic ovary syndrome.
